# Quantum Definition of Molecular Structure

**DOI:** 10.1021/jacs.3c11467

**Published:** 2024-01-10

**Authors:** Lucas Lang, Henrique M. Cezar, Ludwik Adamowicz, Thomas B. Pedersen

**Affiliations:** † Hylleraas Centre for Quantum Molecular Sciences, Department of Chemistry, University of Oslo, P.O. Box 1033 Blindern, 0315 Oslo, Norway; ‡ Technische Universität Berlin, Institut für Chemie, Theoretische Chemie/Quantenchemie, Sekr. C7, Straße des 17. Juni 135, 10623 Berlin, Germany; § Centre for Advanced Study at the Norwegian Academy of Science and Letters, Drammensveien 78, 0271 Oslo, Norway; ∥ Department of Chemistry and Biochemistry, University of Arizona, Tucson, Arizona 85721, United States

## Abstract

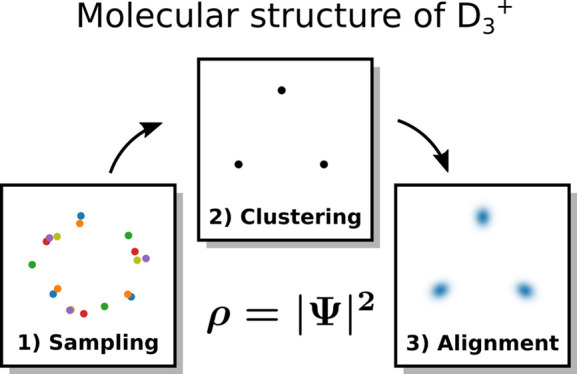

Molecular structure, a key concept of chemistry, has remained elusive from the perspective of all-particle quantum mechanics, despite many efforts. Viewing molecular structure as a manifestation of strong statistical correlation between nuclear positions, we propose a practical method based on Markov chain Monte Carlo sampling and unsupervised machine learning. Application to the D_3_^+^ molecule unambiguously shows that it possesses an equilateral triangular structure. These results provide a major step forward in our understanding of the molecular structure from fundamental quantum principles.

Quantum theory does not admit a rigorous definition of *the* central concept of molecular science: the *structure* of a molecule, i.e., the relative positions of the constituent atoms. While supported by an overwhelming body of empirical evidence, the classical notion of molecular structure is hard to reconcile with the probabilistic nature of the quantum theory of indistinguishable particles. This conflict between the most fundamental concept in chemistry and the thoroughly established physical theory of the atomic-scale world has given rise to debates over several decades.^1−20^ The reconciliation adopted in theoretical and computational chemistry is the Born–Oppenheimer approximation (BOA)^21,22^ which, in essence, *imposes* the molecular-structure concept upon quantum theory by explicitly treating the atomic nuclei as fixed classical point charges, eventually leading to the molecular-orbital theories that elegantly rationalize observed chemical phenomena.^23−25^ The BOA thus is highly successful, and importantly, its mathematical validity and numerical accuracy is very well established.^26^ The fundamental conflict persists, however.

In the past decades, computational techniques have been developed for numerically solving the full molecular Schrödinger equation, treating electrons and nuclei on the same quantum-mechanical footing *without* invoking the BOA at any stage.^27−33^ Several attempts to extract molecular structure from the full wave function have been reported,^34−40^ but so far without unambiguously obtaining the structure of even triatomic molecules.

The full molecular wave function has some peculiarities that might be unfamiliar to many chemists. Since eigenstates of the molecular Hamiltonian must be symmetric or antisymmetric under inversion, the two enantiomers of a given chiral structure have exactly the same probability; i.e., eigenstates for a chiral molecule are quantum superpositions of both enantiomers. This is in conflict with the empirical observation of optical activity, which led Van ’t Hoff to predict the existence of enantiomers as early as 1874. Another complication is the fact that molecular eigenstates are superpositions of different orientations in space of a given classical molecular structure due to rotation.^36^ Cafiero and Adamowicz^36^ attempted to obtain the molecular structure of isotopomers of H_3_^+^, a molecule of great importance in astrochemistry,^41−43^ by computing expectation values of internuclear distances. However, they faced difficulties due to the indistinguishability of identical nuclei: The definition of a molecular structure requires measuring distances between distinguishable particles, e.g., the OH distance in ethanol requires us to be able to distinguish the hydroxy proton from the methyl proton. In quantum mechanics, however, the average OH distance includes the distances of the O nucleus to *all* protons in the molecule (hydroxy, methylene, and methyl). Cafiero and Adamowicz concluded that they cannot distinguish between equilateral triangular and linear structures of the homonuclear H_3_^+^ isotopomers. Only through “isotopic substitution,” considering the HDT^+^ isotopomer where all three nuclei are distinguishable, they were able to confirm the triangular structure that is so straightforwardly obtained within the BOA.^36^

We hypothesize that molecular structure is essentially a manifestation of strong statistical correlation between the positions of the nuclei, which is already an old idea.^5,44^ As such, it should be possible to extract molecular structure information from the joint probability density, i.e., square of the wave function ρ(**r**, **R**) = |Ψ(**r**, **R**)|^2^. Here, **r** and **R** are the internal coordinates of the electrons and nuclei, respectively. Indeed, “elements” of molecular structure have been extracted from the molecular wave function by analyzing reduced densities.^45^ However, using reduced density functions instead of the complete joint probability density represents a loss of information, and it is expected that such an approach would quickly become impractical for larger molecules with many indistinguishable nuclei.

Here, we describe an alternative approach that can distinguish between different structures simultaneously present in a molecular wave function and that directly generates a visualization of the complete molecular structure without any human bias. Furthermore, we will demonstrate that aspects of the electronic structure of a molecule can also be obtained.

We first introduce some preliminary concepts that allow us to define the molecular structure beyond the BOA. The *coordinate vectors* of the nuclei **R** contain subscripts that label the different positions of indistinguishable particles. Furthermore, they correspond to a certain orientation of the molecule in space. Molecular structure is a concept that is invariant under rotations. For example, in a diatomic molecule, the molecular structure is completely determined by the interatomic distance, while the direction of the bond axis in space is irrelevant. We exclude space inversion since different enantiomers are commonly considered to have unique structures. This leads to the definition of *labeled configurations*. A labeled configuration [**R**]^rot^ for a given coordinate vector **R** is the whole set of coordinate vectors that are related to **R** by a simple rotation in space. Similarly, an *unlabeled configuration* [**R**]^rot,perm^ is the whole set of coordinate vectors that are related to **R** by applying a rotation in space *and* a permutation of the positions of identical nuclei. These concepts are illustrated in Figure 1.

**Figure 1:**
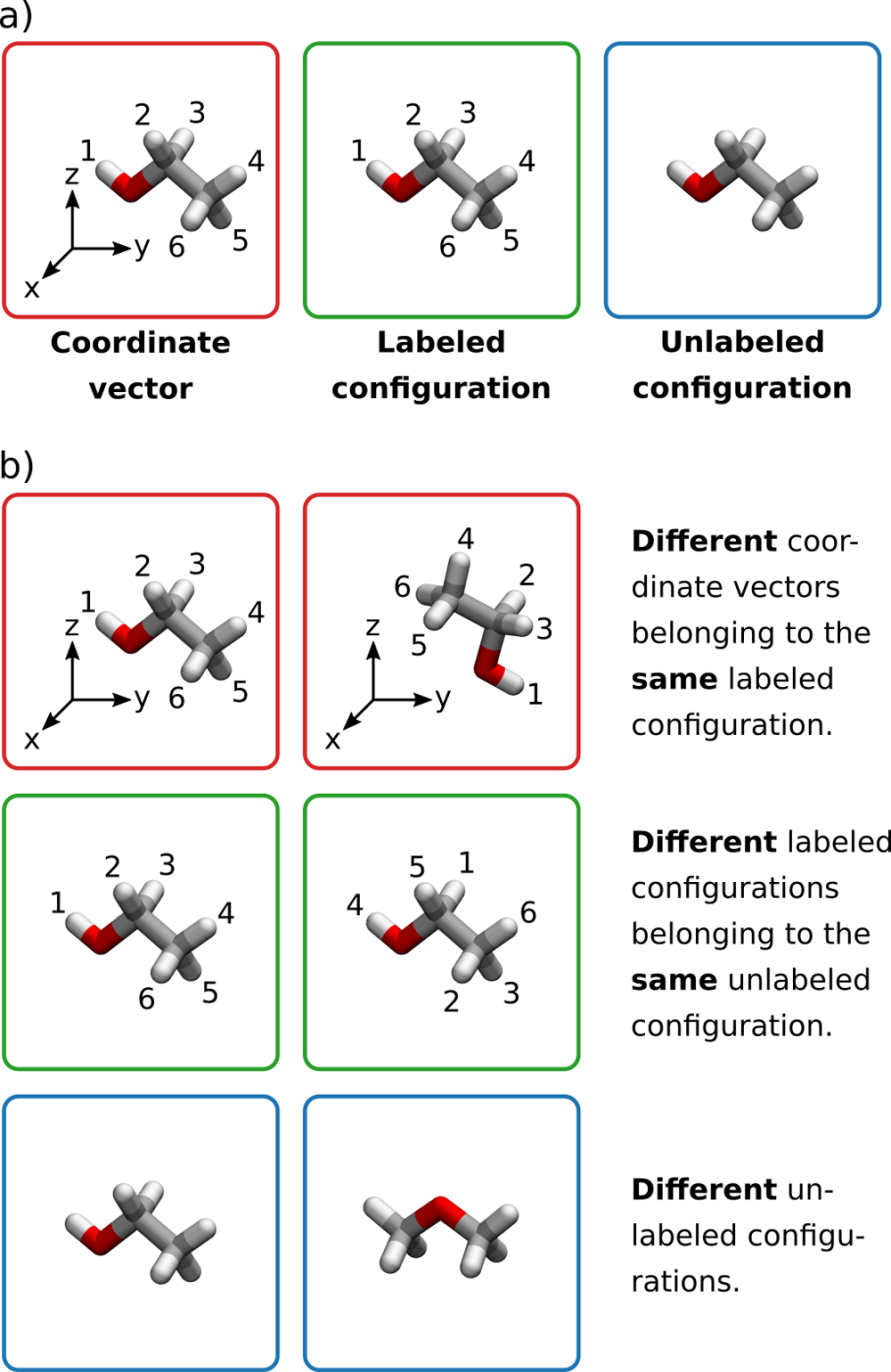
a) Illustration of the concepts “coordinate vector”, “labeled configuration”, and “unlabeled configuration” for molecules with the chemical formula C_2_H_6_O. For coordinate vectors, positions of identical nuclei are labeled and the molecule is oriented with respect to a coordinate frame. For labeled configurations, orientation in space is irrelevant, and for unlabeled configurations also the labels on positions of identical particles are removed. b) Relationships between the three concepts.

Given the nuclear joint probability ρ^nuc^(**R**), the probability density for observing a molecule in a certain labeled configuration is given by a *rotationally integrated* nuclear probability density ρ^nuc,rot^([**R**]^rot^) = *∫*ρ^nuc^(**UR**) d**U**, where the integration is over all possible rotations **U**. Since ρ^nuc,rot^ is invariant under the permutation of positions of identical particles, the probability density for observing an unlabeled configuration is ρ^nuc,rot,perm^([**R**]^rot,perm^) = *N*_perm_ρ^nuc,rot^([**R**]^rot^), where *N*_perm_ is the number of permutations that interchange positions of identical nuclei.

We are now ready to state our new definition of “molecular structure”: A molecular structure is a sufficiently compact high-probability region of ρ^nuc,rot,perm^, i.e., it is a set of unlabeled configurations that are relatively similar to each other and for which ρ^nuc,rot,perm^ assumes relatively large values, such that these unlabeled configurations contribute significantly to the molecular wave function.

In practice, it is convenient to find high-probability regions of ρ^nuc,rot^ instead of ρ^nuc,rot,perm^. If [**R**]^rot^ is a high-probability labeled configuration, then all of the *N*_perm_ labeled configurations obtained by permuting positions of identical particles also have high (namely, the same) probability. Therefore, given a certain high-probability region of ρ^nuc,rot^, there are a total of *N*_perm_ high-probability regions that can be obtained by applying permutation operators. However, not all of these need be unique. If after application of a permutation one obtains a labeled configuration that is similar to the original labeled configuration, then they belong to the *same* high probability region. Such a situation occurs when two nuclei are related by structural symmetry, as illustrated in Figure 2. Therefore, finding high-probability regions of ρ^nuc,rot^ instead of high-probability regions of ρ^nuc,rot,perm^ not only gives us information about the molecular structure(s) dominating a given molecular wave function but also about the molecular symmetry (MS) group.^46^

**Figure 2:**
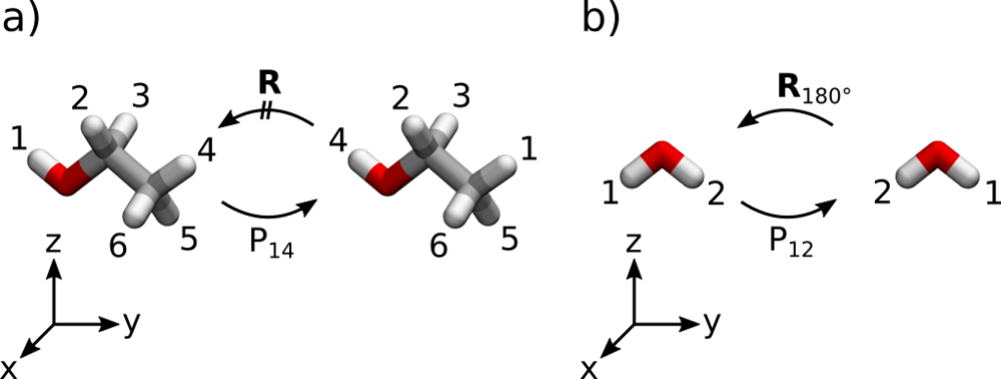
Visualization of identical nuclei related by structural symmetry. a) In ethanol, the permutation *P*_14_ exchanges the labels of the hydroxy proton and a methyl proton. There is no rotation that maps these two protons onto each other and leaves the coordinate vector unchanged, i.e., they are not related by structural symmetry. b) In water, the permutation of the two proton positions can be undone by applying a 180° rotation, i.e., they are related by structural symmetry.

In order to find high-probability regions of ρ^nuc,rot^ in practice, we first draw a sufficiently large random sample from ρ(**r**, **R**). This can be achieved via Markov chain Monte Carlo (MCMC) sampling. Furthermore, a distance metric *d* is required for measuring the similarity of the individual labeled configurations. Two coordinate vectors can be aligned by a rotation such that their root-mean-square deviation (RMSD) in the nuclear center-of-mass frame is minimized. There exist simple equations for finding the optimal rotation matrix **U**_opt_ to achieve this goal.^47^ The metric *d*([**R**]^rot^, [**R**′]^rot^) is defined as the RMSD after alignment. This metric can then be used together with clustering algorithms,^48^ a type of unsupervised machine learning. We choose *k*-medoids clustering,^48^ which represents each cluster by its “medoid”, i.e., the structure that minimizes the sum of distances to all other structures in the same cluster. The only human bias is the choice of the number of clusters *k*. However, there are different heuristics for choosing *k* in an automated manner.^49−51^

Computational details for all of our simulations can be found in the Supporting Information. In order to illustrate our approach, we performed an MCMC simulation for 3 million steps starting from an asymmetric start configuration, based on the joint probability density of a non-BO molecular wave function^52,53^ for the ground state of the D_3_^+^ molecule,^54,55^ one of the homonuclear H_3_^+^ isotopomers studied by Cafiero and Adamowicz.^36^ We chose D_3_^+^ since its ground state, unlike that of H_3_^+^,^32,43^ has total angular momentum *J* = 0,^56^ which is a requirement for our chosen wave function parametrization. In order to demonstrate that the sample size is sufficient, we computed expectation values from the sample and compared them with analytical values; see Table 1. Some snapshots from this simulation are listed in Figure 3. Since the number of distances scales quadratically with the number of data points, the *k*-medoids algorithm cannot be performed on the full set of 3 million snapshots. Therefore, we selected 30000 snapshots with intervals of 100 steps from the simulation and performed *k*-medoids clustering with *k* = 1. Thus, all structures are assigned to the same cluster, and the problem only consists in finding the medoid. The obtained medoid structure is an almost perfect equilateral triangle with a side length of about 1.69 Bohrs (see the Supporting Information for details). This is close to the BO structure having a side length of 1.65 Bohrs. A possible reason for the difference between these two numbers is that our treatment automatically includes vibrational averaging, which typically leads to elongated interatomic distances due to anharmonicity.^57,58^Figure 4 visualizes the results of the clustering. All of the 30000 selected nuclear configurations can be perfectly described with a single cluster, i.e., the chosen value of *k* = 1 is appropriate. Using the terminology of the MS group,^46^ there is only one *version* of the structure since all permutations of the deuterons are *feasible*, i.e., the molecule has the MS group *D*_3h_(M). Furthermore, our analysis shows that the three deuterons in the D_3_^+^ molecule rarely strongly deviate from an equilateral triangular structure.

**Figure 3:**
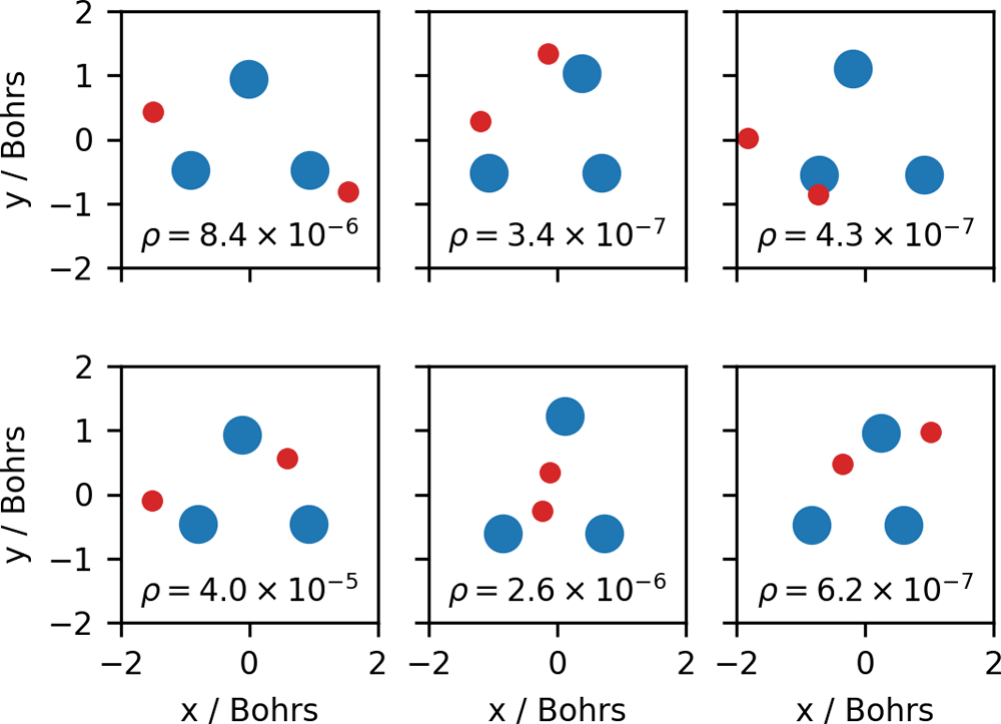
Six snapshots and their ρ(**r**, **R**) values selected from the 3 million step MCMC run after intervals of 500000 steps. The positions are projected onto the plane spanned by the three deuteron positions (blue), which is randomly oriented in space. Electron positions projected onto the nuclear plane are shown in red.

**Figure 4:**
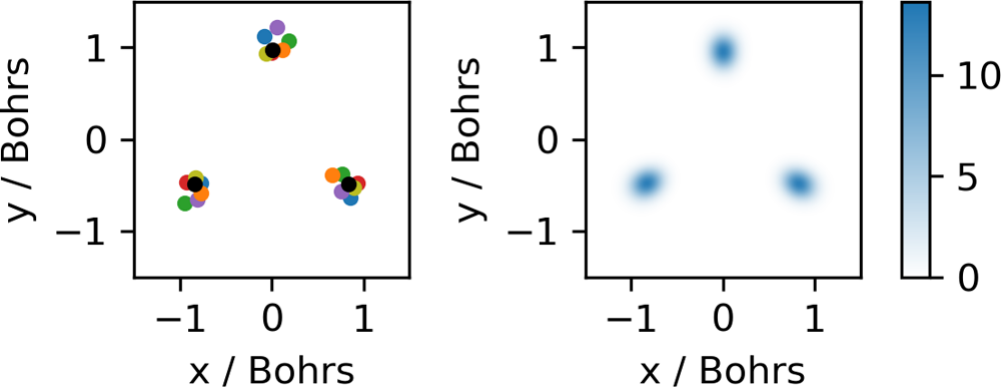
Left: Cluster medoid (black dots) and the configurations of the six snapshots from Figure 3, each plotted in a different color and aligned with the medoid configuration. Keeping in mind the close connection between RMSD minimization and the Eckart frame definition,^59^ deviations from the medoid structure after alignment can be interpreted as resulting from zero-point vibrational motion. Right: 2D kernel density estimate (KDE) of the *x* and *y* values of the deuteron positions of the 30000 selected snapshots after alignment with the medoid configuration.

**Table 1:** Expectation Values Obtained Analytically and from the Random Sample^a^

	⟨*E*_tot_⟩	⟨*r*_DD_⟩	⟨*r*_De_⟩	⟨*r*_ee_⟩
analytical:	–1.328	1.703	1.605	2.021
sample:	–1.329	1.703	1.604	2.018

a ⟨*E*_tot_⟩ was obtained from ⟨*E*_pot_⟩ and the virial theorem. In the interparticle distances, D = deuteron and e = electron. All values are in atomic units (Hartree and Bohr).

We can even obtain information about the *electronic* structure of D_3_^+^. As seen in Figure 3, we are sampling from the full joint probability density, i.e., we also obtain electron positions. After the alignment of all 3 million snapshots with the medoid structure, we obtain an estimate of the electron density by performing a 3D KDE based on the sampled electron positions. A visualization of the obtained density compared with the one obtained within the BOA is shown in Figure 5. Although there are some artifacts from the random sampling in the non-BO density and although the BO density is *not* vibrationally averaged, the agreement between the two treatments is striking.

**Figure 5:**
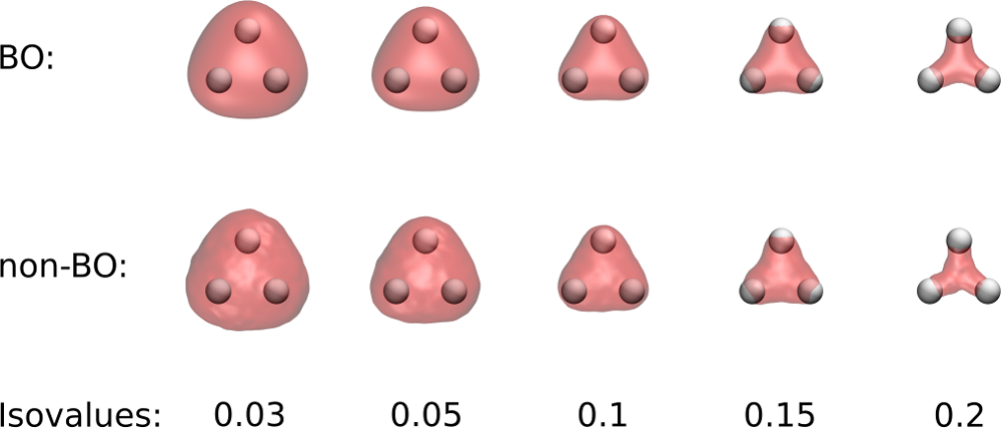
Top: Full configuration interaction electron density and minimum structure. Bottom: Electron density and medoid structure obtained from sampling the non-BO probability density.

In conclusion, we introduced a new definition of molecular structure that is based on the probabilistic interpretation of quantum mechanics and suggested a practical method for obtaining the structure(s) using MCMC sampling and clustering analysis. We applied our approach to the ground state of the homonuclear D_3_^+^ molecule, and demonstrated that our analysis unambiguously yields an equilateral triangular structure very similar to the one obtained within the BOA. Furthermore, we demonstrated that our approach allows a glimpse into the electronic structure of molecules by extracting vibrationally averaged electron densities. The presented methodology will also be applied to excited states in the future. We anticipate that clustering approaches will have the advantage that multiple maxima of the probability density related to wave function nodes of vibrational origin can be assigned to the same cluster. With this powerful new paradigm, we are looking forward to the emergence of future methods that can treat larger molecules in a fully quantum-mechanical fashion and investigate systems such as small chiral molecules, where it is known that both enantiomeric structures must be present with equal probability. In contrast to our interpretation of molecular structure in terms of statistical correlations, understanding the emergence of a purely classical structure, e.g., the fact that chiral molecules exist in unique enantiomers showing optical activity, is still an open problem that remains to be solved.^39^

## Data Availability

The data underlying this study are openly available in Zenodo at DOI: 10.5281/zenodo.8421052. A Snakemake^60^ workflow for reproducing the random sampling and data analysis can be found at https://github.com/LucasLang/molecular_structure_analysis and DOI: 10.5281/zenodo.8425665. It depends on a modified version of the ATOM-MOL-nonBO program^53^ for printing the wave function parameters, which can be found at https://github.com/LucasLang/ATOM-MOL-nonBO and DOI: 10.5281/zenodo.8420768. Furthermore, it depends on our Julia package MolStructSampling.jl, which implements the theory laid out in this article and can be found at https://github.com/LucasLang/MolStructSampling.jl and DOI: 10.5281/zenodo.8413649.

## Supporting Information Available

Theoretical background information, computational details for all simulations reported herein (PDF)
ORCA input file for the full configuration interaction calculation (TXT)
