# Supplementary material for: Quantum Definition of Molecular Structure
Source: J Am Chem Soc. 2024 Jan 10;146(3):1760–4. doi: 10.1021/jacs.3c11467 (PMC10811664; doi:10.1021/jacs.3c11467)
Supplement: Supplementary file 1 — ja3c11467_si_001.pdf [file ja3c11467_si_001.pdf]

Supporting Information: Quantum Definition of Molecular Structure

Lucas Lang,[*,†,‡] Henrique M. Cezar,[†] Ludwik Adamowicz,[¶,§] and Thomas B. Pedersen[†,¶]

†*Hylleraas Centre for Quantum Molecular Sciences, Department of Chemistry, University of Oslo, P.O. Box 1033 Blindern, 0315 Oslo, Norway*

‡*Technische Universität Berlin, Institut für Chemie, Theoretische Chemie/Quantenchemie, Sekr. C7, Straße des 17. Juni 135, 10623 Berlin, Germany*

¶*Centre for Advanced Study at the Norwegian Academy of Science and Letters, Drammensveien 78, 0271 Oslo, Norway*

§*Department of Chemistry and Biochemistry, University of Arizona, Tucson, Arizona 85721, USA*

E-mail: lucas.lang@chem.tu-berlin.de

S1 Theoretical Background

S1.1 Explicitly correlated Gaussian wavefunctions

If $\mathbf{R}_i \in \mathbb{R}^3$ are the Cartesian coordinate vectors of all $N = N_{\mathrm{nuc}} + N_{\mathrm{el}}$ particles (nuclei and electrons) in the molecule, the $n = N - 1$ *pseudoparticle* coordinates are defined as

$$\mathbf{r}_i = \mathbf{R}_{i+1} - \mathbf{R}_1. \tag{S1}$$

Note that in the main manuscript, uppercase and lowercase letters $\mathbf{R}$ and $\mathbf{r}$ refer to nuclear and electronic coordinates, whereas in this supporting information, they refer to particle and pseudoparticle coordinates. Using a spin-free formalism, the internal wavefunction after center-of-mass (COM) separation is a function of those pseudoparticle coordinates and is written as a linear combination (with complex coefficients) of complex explicitly correlated Gaussians (CECGs),[1]

$$\Psi(\mathbf{r}) = \hat{\mathcal{Y}} \sum_{k=1}^{M} C_k \Phi_k(\mathbf{r}), \tag{S2}$$

$$\Phi_k(\mathbf{r}) = N_k \exp(-\mathbf{r}^{\mathrm{T}}(\mathbf{L}_k\mathbf{L}_k^{\mathrm{T}} + i\mathbf{B}_k)\mathbf{r}) = N_k \exp(-\mathbf{r}^{\mathrm{T}}\mathbf{C}_k\mathbf{r}). \tag{S3}$$

Here, $\mathbf{r} = (\mathbf{r}_1, \ldots, \mathbf{r}_n)^{\mathrm{T}}$ and the $\mathbf{L}_k$ are real lower-triangular matrices and the $\mathbf{B}_k$ are real symmetric matrices of dimension $n \times n$. $\mathcal{Y}$ is a product of Young operators that projects the wavefunction onto the correct irreducible representation of the symmetric group and N_k is a normalization constant. The joint probability density for finding certain values of the pseudoparticle coordinates is given by

$$\rho(\mathbf{r}) = |\Psi(\mathbf{r})|^2. \tag{S4}$$

By definition, the pseudoparticle coordinates of the nuclei occur before the those of the electrons in $\mathbf{r}$, the latter of which have the indices from N_{nuc} to n. The nuclear joint probability

density is defined by integrating out the electronic degrees of freedom, i.e.,

$$\rho^{\mathrm{nuc}}(\mathbf{r}_1, \ldots, \mathbf{r}_{N_{\mathrm{nuc}}-1}) = \int \rho(\mathbf{r}) d\mathbf{r}_{N_{\mathrm{nuc}}} \ldots d\mathbf{r}_n. \tag{S5}$$

We do not perform this integration explicitly, but it is important for the conceptual definition of molecular structure given in the main paper.

S1.2 Markov chain Monte Carlo sampling

We draw a random sample from $\rho(\mathbf{r})$ using the Metropolis–Hastings algorithm, a Markov chain Monte Carlo (MCMC) method. New configurations are generated using random displacements of pseudoparticle coordinates. After choosing an initial configuration $\mathbf{r}^{(0)}$, the algorithm iterates through the following steps:

- Randomly choose for which pseudoparticle $i \in \{1 \ldots n\}$ a move is attempted.
- Randomly draw (with uniform probability) a displacement vector $\mathbf{\Delta} \in \mathbb{R}^3$ from a cube with side-length a_i centered around $\mathbf{0}$ and add it to $\mathbf{r}_i^{(t)}$ (the current configuration). This defines the trial pseudoparticle coordinates $\mathbf{r}^{\mathrm{trial}}$.
- Calculate the Metropolis acceptance criterion $A = \min\left(1, \rho(\mathbf{r}^{\mathrm{trial}})/\rho(\mathbf{r}^{(t)})\right)$.
- Draw a random number $u \in [0, 1]$. If $u < A$, accept the move ($\mathbf{r}^{(t+1)} = \mathbf{r}^{\mathrm{trial}}$), otherwise reject it ($\mathbf{r}^{(t+1)} = \mathbf{r}^{(t)}$).

The sampling ends after a predefined number of steps N_s.

S1.3 Expectation values of local operators from importance sampling

For a local operator like the potential energy $V(\mathbf{r})$, a quantum-mechanical expectation value can be written as

$$\langle V \rangle = \int \rho(\mathbf{r}) V(\mathbf{r}) d\mathbf{r}. \tag{S6}$$

Given a random sample $\{\mathbf{r}^{(t)}\}$, e.g. obtained via the MCMC algorithm described in the previous section, the integral can be approximated via importance sampling, i.e.,

$$\langle V \rangle = \int \rho(\mathbf{r}) V(\mathbf{r}) d\mathbf{r} \approx \frac{1}{N_s} \sum_{t=1}^{N_s} V(\mathbf{r}^{(t)}). \tag{S7}$$

This equation was used in order to estimate expectation values of the potential energy and of interparticle distances.

S1.4 Alignment of structures

In terms of pseudoparticle coordinates, the particle coordinates in the nuclear COM frame are given by

$$\mathbf{R}_i^{\mathrm{COM}} = \mathbf{R}_i^{(1)} - \mathbf{R}^{\mathrm{COM}(1)}. \tag{S8}$$

Here,

$$\mathbf{R}_i^{(1)} = \mathbf{R}_i - \mathbf{R}_1 = \begin{cases} 0 & \text{if } i = 1 \\ \mathbf{r}_{i-1} & \text{otherwise} \end{cases} \tag{S9}$$

are the particle coordinates with respect to the reference particle and

$$\mathbf{R}^{\mathrm{COM}(1)} = \frac{\sum_{i=1}^{N_{\mathrm{nuc}}} M_i \mathbf{R}_i^{(1)}}{\sum_{i=1}^{N_{\mathrm{nuc}}} M_i} \tag{S10}$$

is the nuclear COM with respect to the reference particle.

Given two sets of nuclear coordinates $\mathbf{R}_i^{\mathrm{COM}}$ and $\mathbf{R}_i^{\mathrm{COM}\prime}$, *aligning* them means searching

for the rotation matrix $\mathbf{U} \in SO(3)$ such that the RMSD

$$\mathrm{RMSD}(\mathbf{U}) = \sqrt{\frac{1}{N_{\mathrm{nuc}}} \sum_{i=1}^{N_{\mathrm{nuc}}} (\mathbf{R}_i^{\mathrm{COM}} - \mathbf{U}\mathbf{R}_i^{\mathrm{COM}\prime})^2} \tag{S11}$$

is minimized. The optimal rotation matrix can easily be obtained using the method described by Markley.[2] Defining $\mathbf{R}^{\mathrm{COM}}$ as the $N_{\mathrm{nuc}} \times 3$ matrix having the $\mathbf{R}_i^{\mathrm{COM}}$ vectors as rows, one defines the matrix

$$\mathbf{H} = \mathbf{R}^{\mathrm{COM}^{\mathrm{T}}} \mathbf{R}^{\mathrm{COM}\prime} \tag{S12}$$

and obtains its singular value decomposition (SVD),

$$\mathbf{H} = \mathbf{U}\mathbf{\Sigma}\mathbf{V}^{\mathrm{T}}. \tag{S13}$$

Defining $d = \det \mathbf{U} \det \mathbf{V}$, the optimal rotation matrix is given by

$$\mathbf{U}_{\mathrm{opt}} = \mathbf{U} \begin{pmatrix} 1 & & \\ & 1 & \\ & & d \end{pmatrix} \mathbf{V}^{\mathrm{T}}. \tag{S14}$$

The minimal RMSD between the two sets of coordinates is the distance metric introduced in the main paper. This metric depends on the choice of origin of the coordinate system. We choose the nuclear COM as origin since it allows us to interpret variations in the coordinates as vibrations.

S1.5 Kernel Density Estimation

The task in kernel density estimation (KDE) is to find an estimate $\hat{\rho}$ for an unknown probability density function ρ using a random sample $(\mathbf{x}_1, \mathbf{x}_2, \ldots, \mathbf{x}_n)$, with $\mathbf{x}_i \in \mathbb{R}^k$, drawn from

ρ. The estimate is defined as

$$\hat{\rho}_h(\mathbf{x}) = \frac{1}{n}\sum_{i=1}^{n} K_h(\mathbf{x} - \mathbf{x}_i), \tag{S15}$$

where the *scaled kernel* K_h is defined in terms of the *kernel* K and the *bandwidth* h as

$$K_h(\mathbf{x}) = \frac{1}{h} K(\mathbf{x}/h). \tag{S16}$$

The kernel function must be positive and normalized to 1. A common choice often used in practice and also in our present work is the Gaussian kernel given by

$$K(\mathbf{x}) = \frac{1}{\sqrt{(2\pi)^k}} \exp\left(-\frac{1}{2}\mathbf{x}^{\mathrm{T}}\mathbf{x}\right). \tag{S17}$$

S2 Computational Details

For D_3^+, there are $n = 4$ pseudoparticles. The wavefunction is a symmetry-projected linear combination of $M = 400$ CECGs. The wavefunction was optimized by adding CECGs one by one and optimizing their nonlinear parameters variationally. After the total number of CECGs had reached 10, 20, and 400, respectively, the nonlinear parameters of *all* CECGs were variationally optimized one by one multiple times. Since the wavefunction optimization consists of different steps (adding new Gaussians, and optimizing nonlinear parameters), it is difficult to judge the convergence and final accuracy of total energies. The accuracy of the optimized wavefunction was assessed by computing the virial ratio $-2\langle T\rangle/\langle V\rangle \approx 0.999959$, which should be equal to 1 for exact eigenfunctions. The deuteron mass was chosen to be 3670.4829652 atomic units, which agrees with the value 3670.4829678537167 (obtained from the CODATA recommended values for the electron and deuteron mass[3,4]) to nine significant digits. The optimization of the wavefunction parameters was the most computationally expensive step in our work. It was completed on 28 CPU cores in less than one week. The

variationally optimized parameters of the wavefunction are the 400×10 lower-triangular elements of the matrices $\mathbf{L}_k$, the 400×10 lower-triangular elements of the matrices $\mathbf{B}_k$, and the 400×2 real and imaginary parts of the linear expansion coefficients $\mathbf{C}_k$. The total number of real parameters is 8800. The product of Young operators used for symmetry projection is

$$\hat{\mathcal{Y}} = (1 + P_{12})(1 + P_{13} + P_{23})(1 + P_{45}). \tag{S18}$$

Here, P_{ij} stands for the transposition of particles i and j. Particles 1 to 3 are the deuterons, while particles 4 and 5 are the electrons. One should note that the overall wavefunction (including spin) must be totally symmetric for deuterons, which are bosons, and antisymmetric for electrons, which are fermions. The spatial part of the wavefunction for the ground state of D_3^+ is totally symmetric for both deuterons and electrons,[5] which implies that the total deuteron spin is S=1 or S=3 (totally symmetric) and that the total electron spin is S=0 (antisymmetric).

The cube edge lengths a_i for generating random trial moves in the Monte Carlo sampling were chosen as 0.7 Bohrs for pseudodeuterons and 2.2 Bohrs for pseudoelectrons. This led to roughly 50% accepted moves for all pseudoparticles individually, which is the rule of thumb acceptance rate employed in molecular simulations.[6] The asymmetric start configuration for the Monte Carlo sampling is given in Table S1 and shown in Figure S1. In the start configuration, the longest DD distance is almost twice as long as the shortest DD distance, in order not to bias the results toward an equilateral triangular structure. Monte Carlo sampling was performed for a total of 3×10^6 steps. The probability density value of the start configuration

Table S1: Initial coordinates for the Monte Carlo sampling with respect to the first particle D_1 (pseudoparticle coordinates).

	x	y	z
$r_{D_1D_2}$	0.0	0.0	2.3
$r_{D_1D_3}$	0.85	0.0	0.85
$r_{D_1e_1}$	0.0	0.0	1.15
$r_{D_1e_2}$	0.425	0.0	0.425

Table S2: Initial coordinates for the Monte Carlo sampling in the nuclear COM frame.

	x	y	z
$R_{D_1}^{COM}$	-0.2833	0.0	-1.05
$R_{D_2}^{COM}$	-0.2833	0.0	1.25
$R_{D_3}^{COM}$	0.5667	0.0	-0.2
$R_{e_1}^{COM}$	-0.2833	0.0	0.1
$R_{e_2}^{COM}$	0.1417	0.0	-0.625

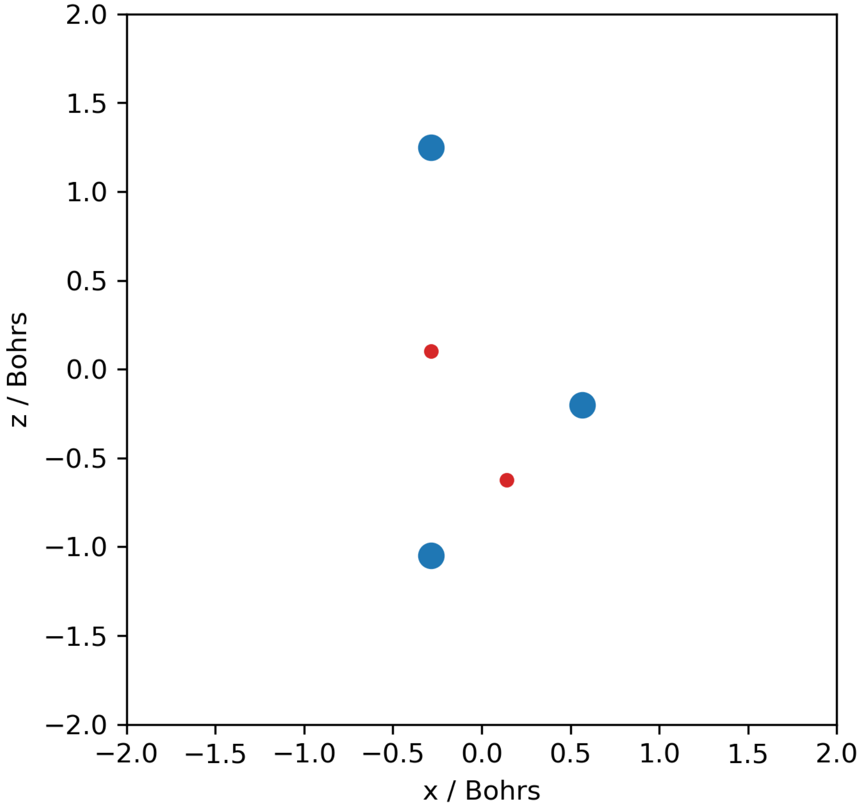

Figure S1: Initial configuration of the Monte Carlo sampling (in the nuclear COM frame, see Table S2). Deuterons are blue and electrons are red.

is $\rho = 3.82 \times 10^{-10}$ and the median value of all 3 million steps is $\rho = 6.21 \times 10^{-6}$. The first probability density value larger than this median was encountered after only 43 steps, i.e., the equilibration period was very short; see Figure S2. Therefore, we included the entire set of sampled configurations in the final analysis.

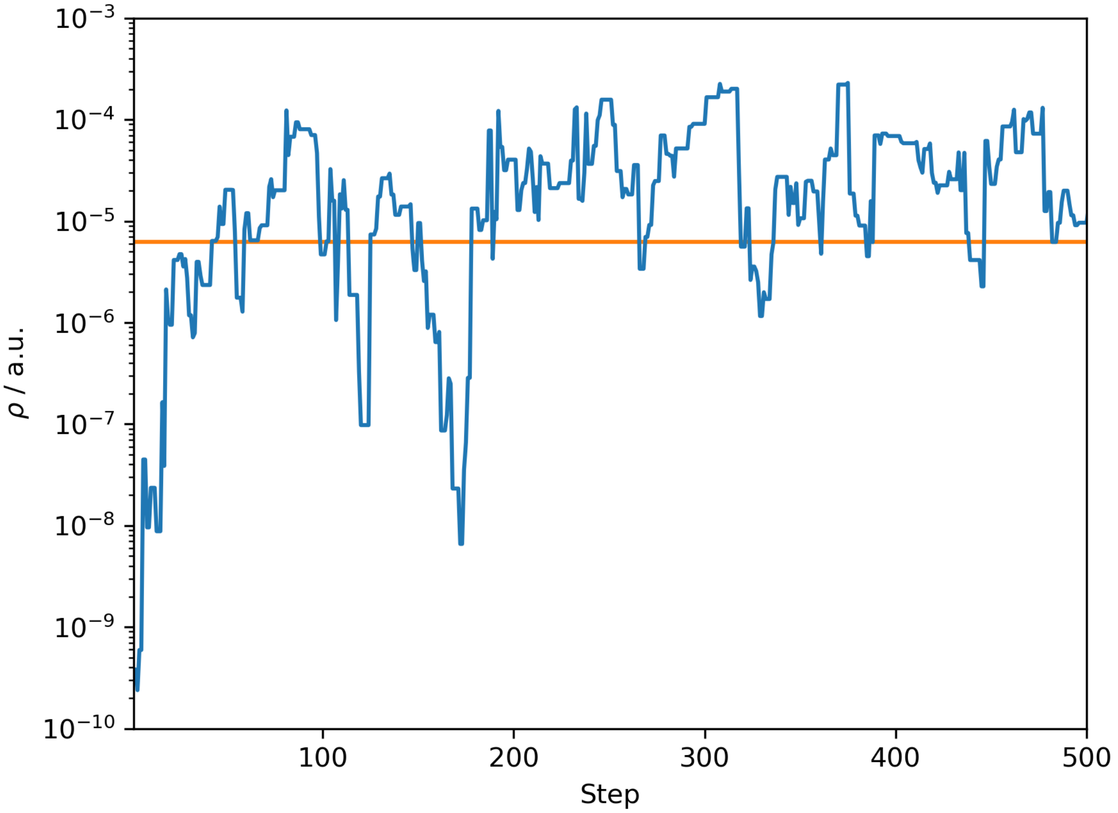

Figure S2: Equilibration of the simulation. The blue line shows the probability density value for the first 500 steps whereas the orange line shows the median value for the entire simulation.

For transforming from pseudoparticle to COM coordinates, the mass of a deuteron was taken to be 3670.4829652 times the electron mass. 30000 snapshots were extracted from the full Monte Carlo simulation after intervals of 100 steps for performing k-medoids clustering. k-medoids clustering was performed with scikit-learn-extra 0.2.0 (https://scikit-learn-extra.readthedocs.io) using a single ($k = 1$) cluster, the `alternate` method and the default `heuristic` initialization method.

In order to generate the KDE estimate of the x and y components of the deuteron positions after alignment with the medoid structure, we used the implementation in scikit-learn[7] 1.2.2 using a Gaussian kernel with a bandwidth of 0.05.

For obtaining the vibrationally averaged electron density estimate, we aligned all 3 million snapshots with the medoid structure and performed a 3D KDE of the electron positions with scikit-learn using a Gaussian kernel with a bandwidth of 0.1. The final density was multiplied by 2 in order to change the normalization to the number of electrons. The full CI minimum structure and electron density were obtained with the CASSCF module of the ORCA quantum chemistry program,[8] using the cc-pVQZ basis set[9] and 2 electrons in 90 orbitals.

The majority of the data analysis was performed with a Snakemake workflow (using Snakemake version 7.25.2) that is openly available at `https://github.com/LucasLang/molecular_structure_analysis`.

The most computationally intensive steps in the post-processing of the non-BO wavefunction were the Monte Carlo sampling, which for 3 million steps took a few hours on a single CPU core, and the calculation of distance matrices for the clustering analysis, which also took a few hours on a single CPU core for each distance matrix calculated on a set of 30000 selected snapshots.

S3 Statistics on the medoid structure

In order to gain an idea for the statistical uncertainty of the obtained medoid structure, we repeated the k-medoids clustering analysis for 10 different subsets of the whole sample consisting of 30000 snapshots each. The interval between snapshots was chosen to be 100 for all 10 subsets, but the first snapshot included in the subset was varied between step 10 and step 100 in increments of 10. The results are shown in Table S3. As can be seen, the value of 1 for $r_{\mathrm{DD}}^{\mathrm{max}}/r_{\mathrm{DD}}^{\mathrm{min}}$ (corresponding to a perfect equilateral triangular structure) lies outside of two

Table S3: Statistics on medoid structures obtained from different subsets of the complete sample. $\bar{r}_{\mathbf{DD}}$ denotes the average of the three deuteron-deuteron distances and $r_{\mathbf{DD}}^{\mathbf{max}}/r_{\mathbf{DD}}^{\mathbf{min}}$ denotes the ratio of the largest and the smallest deuteron-deuteron distance. μ and σ denotes the mean and standard deviation of those two quantities over the ten different subsets. $n = 10$ corresponds to the clustering analysis whose results are reported in more detail in the main paper.

n	$r_{\mathrm{D_1D_2}}$	$r_{\mathrm{D_1D_3}}$	$r_{\mathrm{D_2D_3}}$	$\bar{r}_{\mathrm{DD}}$	$r_{\mathrm{DD}}^{\mathrm{max}}/r_{\mathrm{DD}}^{\mathrm{min}}$
1	1.690	1.696	1.703	1.696	1.008
2	1.701	1.699	1.696	1.699	1.003
3	1.690	1.696	1.704	1.697	1.009
4	1.689	1.700	1.691	1.693	1.006
5	1.684	1.696	1.694	1.691	1.007
6	1.694	1.695	1.686	1.692	1.005
7	1.701	1.692	1.692	1.695	1.005
8	1.698	1.697	1.700	1.699	1.002
9	1.680	1.691	1.694	1.688	1.008
10	1.687	1.696	1.687	1.690	1.005
μ				1.694	1.006
σ				0.004	0.002

standard deviations from the mean. However, since the mean and standard deviations are estimated from only 10 data points, these quantities are relatively uncertain. Furthermore, the distribution of $r_{\mathrm{DD}}^{\mathrm{max}}/r_{\mathrm{DD}}^{\mathrm{min}}$ values cannot be normal, since the smallest possible value is 1. Finally, the 10 different selections are probably not entirely uncorrelated. For these reasons, the statistics are not perfectly conclusive. The clustering analysis would have to be repeated with a larger number of snapshots in order to show without doubt whether the "true" medoid structure (i.e., the one obtained in the limit of an infinitely large sample size) is exactly equilateral triangular.

References

(1) Bubin, S.; Adamowicz, L. Matrix elements of N-particle explicitly correlated Gaussian basis functions with complex exponential parameters. *J. Chem. Phys.* **2006**, *124*, 224317.

(2) Markley, F. L. Attitude Determination Using Vector Observations and the Singular Value

Decomposition. *J. Astronaut. Sci.* **1988**, *36*, 245–258.

(3) CODATA Value: electron mass. https://physics.nist.gov/cgi-bin/cuu/Value?me, Accessed: 2023-11-18.

(4) CODATA Value: deuteron mass. https://physics.nist.gov/cgi-bin/cuu/Value?md, Accessed: 2023-11-18.

(5) Alijah, A.; Wolniewicz, L.; Hinze, J. Rotation-vibrational states of D_3^+ computed using hyperspherical harmonics. *Mol. Phys.* **1995**, *85*, 1125–1150.

(6) Swendsen, R. H. How the maximum step size in Monte Carlo simulations should be adjusted. *Phys. Procedia* **2011**, *15*, 81–86.

(7) Pedregosa, F.; Varoquaux, G.; Gramfort, A.; Michel, V.; Thirion, B.; Grisel, O.; Blondel, M.; Prettenhofer, P.; Weiss, R.; Dubourg, V. et al. Scikit-learn: Machine Learning in Python. *J. Mach. Learn. Res.* **2011**, *12*, 2825–2830.

(8) Neese, F. Software update: The ORCA program system—Version 5.0. *Wiley Interdiscip. Rev.: Comput. Mol. Sci.* **2022**, *12*, e1606.

(9) Dunning, Jr., T. H. Gaussian basis sets for use in correlated molecular calculations. I. The atoms boron through neon and hydrogen. *J. Chem. Phys.* **1989**, *90*, 1007–1023.
